# Introduction to the Issue: Advances in Theory and Research on Multiple Code Theory and the Referential Process

**DOI:** 10.1007/s10936-025-10190-0

**Published:** 2026-01-17

**Authors:** Wilma Bucci, Henry Peterson

**Affiliations:** 1 Derner Institute, Adelphi University, 1 South Ave, Garden City, NY 11530 USA; 2 The City College of New York, CUNY, New York, USA

**Keywords:** Referential process, Psycholinguistics

## Abstract

This issue is based on a conference that was held in July 2023 and that focused on new advances in the theory and measures of the Referential Process (RP). The concept of the RP was developed in the context of the Multiple Code Theory (MCT) and concerns the complex process by which people are able to connect all manner of experiences, including bodily and emotional experience, to words, in both spoken and written language. A previous issue of this journal, published in 2021, outlined the theory of the referential process, and included empirical and clinical applications. The research studies featured measures of language style based on the Discourse Attributes Analysis Program (DAAP), a computerized text analysis system that was developed by Bernard Maskit in the context of MCT. The 2023 conference covered new developments in the DAAP research program and also featured Dr. Maskit’s presentation of the new TDAAP (Time-based DAAP) which analyzes spoken language on the basis of time flow rather than word count. This new program takes a major step towards examining the role of sensory and bodily experience in the verbal communication of experience. The conference was sponsored by the Pacella Research Center of the New York Psychoanalytic Society and Institute. It included both in-person and online participants across the U.S. and in Canada, Israel, and Italy, and represented the combined efforts of researchers and clinicians. At the conclusion of the conference, the editor of this journal, Dr. Rafael Javier, invited submission of the papers presented at the meetings for a special issue of the journal. In this introduction, we will briefly outline the theory that provides the conceptual framework for our projects. The next section will provide a brief introduction to the papers that are included in this issue.

## Introduction

This issue is based on a conference that was held in July 2023 and that focused on new advances in the theory and measures of the Referential Process (RP). The concept of the RP was developed in the context of the Multiple Code Theory (MCT) and concerns the complex process by which people are able to connect all manner of experiences, including bodily and emotional experience, to words, in both spoken and written language. A previous issue of this journal, published in 2021, outlined the theory of the referential process, and included empirical and clinical applications. The research studies featured measures of language style based on the Discourse Attributes Analysis Program (DAAP), a computerized text analysis system that was developed by Bernard Maskit in the context of MCT.

The 2023 conference covered new developments in the DAAP research program and also featured Dr. Maskit’s presentation of the new TDAAP (Time-based DAAP) which analyzes spoken language on the basis of time flow rather than word count. This new program takes a major step towards examining the role of sensory and bodily experience in the verbal communication of experience. The conference was sponsored by the Pacella Research Center of the New York Psychoanalytic Society and Institute. It included both in-person and online participants across the U.S. and in Canada, Israel, and Italy, and represented the combined efforts of researchers and clinicians. At the conclusion of the conference, the editor of this journal, Dr. Rafael Javier, invited submission of the papers presented at the meetings for a special issue of the journal.

In this introduction, we will briefly outline the theory that provides the conceptual framework for our projects. The next section will provide a brief introduction to the papers that are included in this issue.

## The Challenge of Expressing Experience in Language

The process of expressing experience in words, and the difficulties inherent in this process, can be observed in a wide range of areas—in the arts and sciences, in sports, in the everyday tasks of life, and perhaps most obviously, in psychotherapy. It is a challenge to describe what is expressed in a painting, or a string quartet; it is also a challenge to describe how to enter a highway or change lanes, how to improve a serve in tennis or how to cook pasta to exactly the right point. Such functions are more effectively demonstrated than described in words. A verbal description may accompany the demonstration to help direct understanding, and to guide future use, but the skills need to be shown and modeled, and new networks of knowledge in sensory and motoric systems need to be built.

Expressing emotion verbally presents a particular challenge; this difficulty is outlined by the philosopher Susanne Langer:

Everybody knows that language is a very poor medium for expressing our emotional nature. It merely names certain vaguely and crudely conceived states, but fails miserably in any attempt to convey the ever-moving patterns, the ambivalences and intricacies of inner experience, the interplay of feelings with thoughts and impressions, memories and echoes of memories, transient fantasy, or its mere runic traces …^ (Langer, 1942, p. 100).

The singer known as Iggy Pop focuses on somatic and sensory aspects of his experience in the song ‘I can’t explain’:

Got a feeling inside (Can’t explain).

It’s a certain kind (Can’t explain).

I feel hot and cold (Can’t explain).

Yeah down in my soul, yeah (Can’t explain).

I said (Can’t explain).

I’m feeling good now, yeah but (Can’t explain).

Dizzy in the head, and I’m feeling blue.

Poets and novelists spend their working lives struggling with the difficulty of finding the right words in their own writing; they have in some cases described the difficulty by attributing it to their characters. In her novel, *What Did it Mean*, the writer Angela Thirkell empathizes with Lydia Merton’s struggles with her feelings, and tries to describe a process that seems to be related to the process we are investigating in our work:

“.. she was at the same time conscious of a small dull ache of resentment inside herself; the constriction that the less explored windings of our strange minds send to our equally peculiar bodies long before we know why we suddenly feel hollow and knotted inside…there would appear to be no way of short-circuiting the horrid connection, swifter than lightning or super-sonic something-or-other, between head and whatever that bit of our insides is that takes the fatal electric shock, the knockout blow. (Thirkell, 1954, p. 126)

Tolstoy’s Anna Karenina struggles to understand not only what she is feeling but also how she is thinking:

She felt at that moment that she could not put into words the sense of shame, of rapture and of horror at this stepping into a new life, and she did not want to speak of it, to vulgarize sthis feeling by inappropriate words. But later too, and the next day and the third day, she still found no words in which she could express the complexity of her feelings; indeed she could not even find thoughts in which she could clearly think out all that was in her soul.^ (Tolstoy, 2000 (1878), p. 150).

When Anna sought to find thoughts, she was presumably seeking some kind of words other than the labels ‘shame, rapture, horror’ that Tolstoy provides. Perhaps to some extent she was avoiding such thoughts but was also struggling to find them.

## Other Areas of Work on Generating Language

To our knowledge, the difficulties involved in connecting experience to words do not seem to be recognized within cognitive science or psycholinguistics, and the processes applied in making such connections in and around the act of speaking or writing have not been studied outside of our own work. This contrasts sharply with the extensive work that has been done on two other major contexts, evolutionary and developmental, of the emergence of language.

There is an extensive and expanding – but still inconclusive - literature on how language emerged in the human species (Bickerton, 1990; Hauser et al., 2014). The lack of agreement by scientists in this area is not surprising for a function that is generally estimated to have appeared somewhere between fifty and one hundred thousand years ago. The answer seems to reside in some combination of physiological structure and social and physical environment, but the problem continues to elude scientists. Nevertheless, the field of research remains active in diverse areas, including comparative animal behavior, paleontology, archaeology, and molecular biology, as well as linguistics. New methods of study are continually being developed in these areas, with as yet considerable disagreement as to procedures, and without clear results.

There is also a large field of active research, with clearer findings, concerning the development of language in children. The success of this work and the agreement (with some exceptions) among researchers can be attributed to the opportunity to observe this development as it occurs, in contrast to the prehistoric period of the initial emergence of language. Many graduate students began their careers by installing tape recorders in the rooms of their infant children and developing theories to account for their observations. Over the years, a wide range of sophisticated methods for tracking the development of language in the early months of life has developed. The interaction of linguistic theory and sophisticated methods of observation has led to increased knowledge concerning child language development, and new questions are continually being addressed. There is now considerable agreement that the complex set of functions underlying language development build gradually towards the end of the first year of life (Tomasello & Akhtar, 2000). There is also evidence that understanding verbal communication in the early months of life depends more on the prosodic forms of language than on semantic contents (Fernald, 1989, 1993).

In contrast to the extensive research in these fields, there has been little attention to the process of connecting experience and language *in the moment of speaking or writing*. As Bucci has suggested, this may reflect the assumption, still widely held in cognitive science, psycholinguistics, and related fields, that mature adult thought is by its nature verbal (or in some related symbolic propositional code) and can be communicated directly. (See Bucci, 1997, 2021 for a review of these alternative characterizations concerning the forms of thought).

A contrasting view of mental functioning, termed ‘Parallel Distributed Processing’ (PDP), was offered by Rumelhart et al. (1986), introducing the notion of subsymbolic processing as the basis of mental organization. The PDP modality includes “representations and processes in which the elements are not discrete, organization is not categorical, processing occurs simultaneously in multiple parallel channels, higher level units are not generated from discrete elements, and explicit processing rules cannot be identified.” (Bucci, 1997, p. 88). The introduction of the PDP models was groundbreaking in its recognition of systematic human information processing outside of the verbal, symbolic mode. However, the researchers in that field did not resolve the problem of connecting the subsymbolic PDP models, which were highly specialized for particular tasks, to the symbolic functions that underly language production. The field was left with the same question that we are addressing with our work – how to connect the flow of experience, on multiple simultaneous levels, to the discrete single channel of verbal language. (We note that the new world of Chatbots does not address this process; the language of Chatbot is generated by analyzing vast amounts of data *that have already been produced in text form*).

Our project has been to develop a theoretical framework, with accompanying methods and measures, to address the question of how we connect experience and language. In some ways similar to the field of language development, the work is made possible by the availability of contexts of observation. The basic foundation of the work has been the interaction of theory and measurement. The interactions of psychotherapy have provided one highly relevant and generally accessible area of observation (with sufficient safeguards to protect the privacy of participants). The theory has provided the context for the development of the measures, and these have guided observation; observation has, in turn, fed back into the evaluation and expansion of the theory.

## The Theoretical Framework of MCT and the RP

As Bernie Maskit notes in his paper (this issue), Wilma Bucci and colleagues took the first steps toward addressing how we connect experience and words, leading eventually to developing the concept of the Referential Process (RP) in a series of papers beginning in 1978. In these studies, they identified a relationship between features of language style and the use of hand movements linked to rhythm and intonation patterns of speech. They found that speakers who produced language that was rated as more specific and vivid, and with more imagery, were significantly more likely to accompany their speech with rhythmic speech-related hand movements (Bucci & Freedman, 1978; Bucci, 1984). They also found that silences prior to, or intervening in speech, were associated with particular types of body movements, involving both energy discharge and self-stimulation. Different types of body movements were associated with different functions of silence, such as periods of search and periods of reflection, and were also associated with qualities of the narratives that were produced (Gilani et al., 1985).

Bucci continued to examine the relation of language to other processing systems, focusing on psychotherapy, and using concepts of cognitive science to develop a psychological model of the psychoanalytic process. At that time, in the so-called ‘cognitive revolution,’ the field of cognitive science itself was moving from an assumption that all mental functions were registered in verbal form to a recognition by some scientists that information could be stored and processed in the form of imagery. Based on the dual coding theory of Paivio (1971), as well as work by Bower (1981), Kosslyn (1975, 1980), and others, Bucci (1984) introduced the Dual Code Model as a theoretical framework for the organization of information in memory.

In recognition of continuing advances in cognitive science during the subsequent decade, particularly in the work on the PDP paradigm, as noted above, Bucci developed the multiple code theory (MCT), which incorporated a distinction between nonsymbolic and symbolic representations, including both imagery and language (Bucci, 1997). While the dual code model took a first step toward accounting for the difficulties of expressing experience in language, the multiple code theory greatly expanded our recognition of the complexity and difficulty of this connecting process. According to multiple code theory, based on current research in cognitive science, both nonsymbolic and symbolic forms of representation and communication are present from the beginning of life; both forms change in complexity throughout life; both may be processed within or outside of awareness.

## The Theory of the Referential Process

The fundamental differences in format of representation of subsymbolic (or nonsymbolic) and symbolic processes underly the difficulties in connecting these formats that have been described and illustrated earlier in this paper. All of these formats may operate within or outside of awareness. We have identified three functions in this connecting process: *Arousal*,* Symbolizing/Narrative*, and *Reflecting/Reorganizing*, based primarily on the degree to which one of the formats of representation outlined in MCT is dominant within one’s awareness.

*Arousal*:Nonsymbolic functions are largely dominant in the examples of the difficulties of expressing emotions given earlier. Iggy is feeling hot and cold, dizzy and blue; Thirkell’s character, Lydia Merton, is “conscious of a small dull ache of resentment inside herself;” Tolstoy’s Anna is all too aware of a feeling that is precious to her, that she does not want to vulgarize by inappropriate words. (Tolstoy provides the labels *shame*,* rapture*,* horror;* they are not Anna’s words). All of these are examples of how people try to talk about what they are experiencing when the flow of nonsymbolic functions is dominant in experience.

*Symbolizing/Narrative*: The gift of great writers or powerful speakers is to describe images and tell stories that carry emotional meaning and that have the power to evoke corresponding experiences in a listener or reader. In MCT, the nature of this process is defined as depending on the operation of *emotion schemas* (Bucci, 2021). These are clusters of feelings, including nonsymbolic somatic and sensory functions, that have been activated during emotionally charged events of a person’s life. A writer or speaker can activate the flow of feelings associated with a particular emotion schema by describing such associated events in concrete and specific detail. Anna cannot do it; Tolstoy does it in a very long novel.

*Reflecting/Reorganizing*: Once the speaker has brought to mind and described an event related to an emotion schema, then it may be possible to find emotional meaning in the experience - sometimes new emotional meaning that has not previously been recognized. We can see this function occasionally, though not often, in literature. The reflective function is most salient in psychotherapy, as will be discussed. Most poets and writers of fiction focus on the image or the narrative and leave the reflection largely to the reader – or to the critics.

## The Referential Process in Psychotherapy

The central role of emotion schemas and the functions of the referential process can be seen most directly in psychotherapy, particularly psychoanalysis and psychodynamic therapy. Many of the papers in this issue focus on that setting. From a theoretical perspective, psychoanalysis and cognitive science remain distant, with neither field generally acknowledging the value of the other (Bucci, 2000). On the other hand, the recognition of different modes of experience and the difficulties connecting them, a central premise of MCT, is also a foundational premise of psychoanalytic theory, expressed in different forms, and at different stages in Freud’s development of the theory: in the concepts of the *primary and secondary processes*, defined initially in terms of psychic energy; in the distinctions among *unconscious*,* preconscious*,* and conscious modes of thought* in the topographic model; and in the distinctions among *id*,* ego*,* and superego* (in the structural model).

The concept of the primary process has been associated with both unconscious processes and the id; in his later years, Freud attempted to reconcile these models. In ‘Outline of Psychoanalysis’ he wrote:

We have found that processes in the unconscious or in the id obey different laws from those in the preconscious ego. We name these laws in their totality the primary process, in contrast to the secondary process which governs the course of events in the preconscious, in the ego. In the end, therefore, the study of psychical qualities has after all proved not unfruitful. [Freud (1940, p. 164)]

While Freud’s model of mind incorporates several distinct systems of functioning and different ‘psychical qualities,’ he does not address the question of *connecting* these systems. Instead, the goals of psychoanalytic treatment are generally defined as *translating* or *replacing* one system with another: ‘making the unconscious conscious’; or ‘where id was, there ego shall be.’^1^

As Jaffe notes in his paper in this issue, a group of psychoanalytic thinkers recognized related problems in Freud’s various models, and in their application to treatment, particularly in accounting for the concept of ‘working through’; John Gedo was one of these. Gedo’s thinking on this topic was in agreement with aspects of the theory of the referential process. In his 1995 paper, “Working Through as Metaphor and as a Modality of Treatment,” Gedo writes:

Mental activities Freud (1911) called the primary process become operational earliest; the secondary process becomes additionally available once the child’s symbolic capacities mature. The third phase in the development of thinking is acquisition of the ability to correlate the two processes—a third process Bucci (1993) calls “referential activity.”.. The talent for finding meanings in the data produced by free associations is a function of the degree to which referential activity has become elaborated. In other words, for psychologically obtuse persons, the process of working through necessarily involves the expansion of referential activity^2^—that is, the actual reorganization of the relevant aspects of brain function. (Gedo, 1995, p. 351)

While Gedo recognized the need to connect the primary and secondary processes as part of the process of working through in treatment, he seemed to restrict this need to people he characterized as ‘obtuse.’ As we emphasize in our work, the challenges involved in ‘the ability to correlate’, i.e. to connect nonsymbolic and symbolic processes, are not restricted to ‘obtuse’ persons, and are not restricted to psychotherapy, but occur throughout life, for all people, in a wide range of activities.

## The Organization of Memory and the Role of Awareness

Our experience, in all forms of representation, is entered into the networks of memory in what has been termed the ‘normative unconscious,’ and is retained to varying degrees, and retrievable with varying degrees of effort. We can process symbolic functions out of awareness, as anyone who has gone to sleep with a specific problem and woken up several hours later with a new idea knows. We have also been quite aware of nonsymbolic sensory and somatic experiences that may be highly pleasurable or highly painful and may be in the foreground of experience as well as in the background.

Here, we may briefly contrast the psychoanalytic assumptions concerning the organization of memory with the multiple code view. The psychoanalytic claim is that some painful and threatening experiences – those classified as ‘repressed’ in the classical psychoanalytic model – may be retained in memory in unconscious and inaccessible form. These would be the types of memories that form the latent content of dreams, jokes, or slips of the tongue, or that were viewed as underlying the physical symptoms of hysteria. From the classical psychoanalytic perspective, successful treatment leads to retrieval and reporting of these latent contents; this process will bring about the cure that is sought.

On the other hand, in current views, memory is not a fixed storage system but a constantly changing and interacting set of networks (Damasio, 1994; Phelps, 2006; Pessoa, 2008). This view has also been held in various forms within cognitive science, at least since the time of Bartlett (1932). The networks that make up the emotion schemas include all types of sensory and somatic experiences, as well as conceptual contents. The problems that bring patients to treatment involve painful experiences that lead to distortions in beliefs and expectations concerning the physical and interpersonal worlds; people will try to avoid contexts that activate painful schemas. The painful underlying emotion schemas are constantly being changed to include not only the initial painful events and those related to them, but attempts at self-protection, and its aftermath, throughout life. From this perspective, treatment involves activation of a painful emotion schema in a new interpersonal context, one in which expectations and beliefs about other people – and about oneself - can gradually be changed. The patient tells stories and describes images associated with the emotion schema; the new interactions with the therapist can potentially be incorporated in the emotion schema that has been activated and bring about change.^3^

## Development of Measures of the Referential Process

The central strength of our project has been the continuing interaction between the application of theory and the development of measures adequate to address research questions that evaluate and expand theory. In his talks at the July 2023 conference, as presented in this issue, Maskit focused on this interaction. Based on the theory of the referential process, Maskit and colleagues developed new measures that led to a new understanding of the functions of the process. We briefly summarize the pathways to the new measures in order to emphasize some of the innovations that have been made possible by this interactive research approach.

The function of Narrative/Symbolizing was initially assessed by judges rating segments of text for Referential Activity (RA) based on qualities of language style, and following procedures outlined in a manual. The RA scales provide a measure of the *style of language* when people are describing imagery or telling stories and apply across a wide range of contents. Based on the judges’ ratings, a first computerized measure of the Narrative/Symbolizing function, the CRA, was developed (Mergenthaler & Bucci, 1999). Using a different approach, Maskit then developed a weighted dictionary of the symbolizing function, the Weighted Referential Activity Dictionary (WRAD), which had higher coverage and greater reliability than the earlier versions and also enabled production of derived measures that provided new and previously unavailable information about language style. About 80–85% of the words (tokens^4^) in most spoken discourse are matched by an item in the WRAD. The applications made possible by the new derived measures are described in Maskit’s papers and also shown in many of the papers included in this issue.

The first computer measure of Reflecting/Reorganizing (the REF) was an unweighted word list that had been developed using standard procedures of computer assisted content analysis in which judges selected words that referred to cognitive or logical functions. As Maskit (2021) noted, there is evidence that the REF is associated with the Reflecting/Reorganizing function, as shown by measures of validity, but the coverage is far lower than the WRAD, ranging between 8 and 10% of words in texts such as therapy sessions. This is actually relatively high for unweighted dictionaries, but is far lower than the coverage achieved by the WRAD and is not suitable for production of the derived measures.

As Maskit described in his presentation at the conference, as edited for this issue, a crucial feature in the design of the WRAD was that the word lists were developed by judges rating their reactions to whole passages, rather than through ratings of individual words, as is customary for development of computer measures. He then proposed the development of a weighted computerized measure of the Reflecting/Reorganizing function that was similar to the basic approach used for the WRAD. This involved rating segments of text for qualities of language style associated with the Reflecting/Reorganizing function as had been done for the Narrative/Symbolizing function, rather than ratings of individual words. Once this was done, a weighted dictionary could be constructed that had greatly increased coverage and could be suitable for the construction of derived measures.

The problems with applying this proposal, contrasting with the development of the WRAD, were that *the qualities of language-style associated with Reflecting/Reorganizing had never been identified*, *and no rating manual had previously been developed*. In other words, Maskit was proposing that we now somehow replicate a process that had been carried out over a decade or more in a relatively short span of time. The members of the Referential Process Research Group, mainly post-doctoral fellows and graduate students, as well as Bernie Maskit and Wilma Bucci, recognized the value of Maskit’s vision (with some reservations as to its feasibility), but were eventually quite successful in meeting this challenge. In carrying out this project, the members of the group also developed a new and broader understanding of the Reflecting/Reorganizing function as it occurs in thought and in treatment. The development of the new WRRL (the Weighted Reflection/Reorganization List) is described by Zhou et al. (2021). Similar procedures were then applied in developing the WRSL (Weighted Arousal List), as discussed by Tocatly (this issue). The application of these measures may be seen in the papers included in this issue, and the special issue from 2021.

## Incorporating time

As presented earlier in this paper, and as Maskit also discussed, the origins of multiple code theory were in the early observations of relationships among body movements, speech rhythms, and qualities of language. While the weighted language measures and the derived measures based on them had been developed in the context of the theory, the measures themselves were based only on words. The only partial indicators of speech rhythms that were available in our measures were a few spoken markers of hesitation that were included in the Disfluency dictionary.

It was obvious from the beginning of our work that we were losing considerable information concerning the processes involved in generating language and the interactions among speakers by relying on measures based on words alone. This was particularly apparent in our studies of psychotherapy. In the first of a series of novels by the clinical psychologist, Frank Tallis, set in turn-of-the-century Vienna and featuring a crime investigator who is a follower of psychoanalytic ideas, a fictional Dr. Freud explains his doubts concerning the effectiveness of hypnosis and advocates the new method of free association:

‘As you know,’ continued Freud, ‘I have abandoned hypnosis in favour of free association – encouraging the patient to say whatever comes to mind, without censorship. The analyst listens, and learns not only from what is said but also from its character and form: the silences, the hesitations, the changes of volume and direction. (Tallis, F. p. 282).

The development of the TimeDAAP was the last project that Bernie Maskit was engaged in, and is described in some detail in his presentation. We expect to move forward with applications of this and related programs in our ongoing work. We expect that the introduction of the time dimension will reveal a more complex and multi-layered picture of the referential process as it plays out both within, and between, individuals.

## An Overview of the Contents of the Issue

As mentioned previously, the bulk of this issue consists of papers written by colleagues invited to present at the Referential Process Conference in the summer of 2023. In the months following Dr. Javier’s offer to gather these papers into a special issue, the issue underwent changes in its organization; after the death of Bernard Maskit, in March 2024, Dr. Javier and the members of the Referential Process Research Group decided it should be dedicated to his memory. In addition to the change of title, various colleagues of his were asked to write reflections on Maskit’s legacy. The first two sections are comprised of papers that detail this legacy, his various achievements within the context of the development of the Discourse Attributes Analysis Program (DAAP), and the related measures as applied to the study of the Referential Process. Subsequent sections are based on work presented at the Referential Process Conference in July 2023.

The first section, *Reflections on the Legacy of Bernard Maskit*, includes three essays that speak, not only to his intellectual accomplishments, but to the sense of generosity and warmth that suffused his interactions with others. Negri writes that Maskit was a truly transdisciplinary thinker, a quality that allowed him to address the challenges of mathematics and empirical measurement from a third, conceptual territory. Murphy provides a moving reflection on his time working with, and learning from, Bernard Maskit, painting a portrait of a generous collaborator, expert problem-solver, and consummate teacher. In remembering his years with Maskit, Hoffman reflects on how the beautiful curves of Maskit’s graphs are a gateway to understanding how we translate discrete pieces of information, like words, into the music of continuous experience.

The next section, *Presentation of Core Concepts*, begins with a lightly edited version of several talks that Maskit gave at the 2023 conference. These outline the operation and development of the automated measures of language style, including a first presentation of the new version of Maskit’s program based on the dimension of time—the Time-DAAP—rather than on individual words. Perry Susskind, PhD, a mathematician, and former student of Maskit’s, has contributed a paper on the mathematical foundations of Maskit’s development of the measures and the text analysis program. As Susskind notes, the particular combination of mathematical expertise and creativity that marked Maskit’s thinking produced a variety of unforeseen discoveries when applied to questions of empirical measurement. His creation of the smoothing function, enabling graphic representations depicting the flow of discourse, also enabled a point-by-point comparison between a graphical representation of the functions and the words of a corresponding text. The readers of this issue, who are mainly psychologists and psycholinguists, rather than mathematicians, are confronted with questions of how or why some combination of weighting functions and other mathematical processes enabled a new understanding of the psychological processes they operationalized. We believe the papers in this issue begin to address these questions.

The multiple code theory, and the associated theory of the referential process, have been in dialogue with fields of both linguistic theory and psychoanalytic thought since their inception. While the theories are applicable to a range of domains, and are grounded in the field of cognitive science, many questions remain unresolved. The issue treats aspects of this relationship in its next section, *Advances in Theory*, with papers on the epistemological foundations of the discourse measures (Negri & Barazzetti), and a paper relating the multiple code concept of the emotion schemas to the psychoanalytic concept of unconscious defense (Hoffman).

The remainder of the issue is concerned with empirical applications of the measures, including both process and outcome studies, and microprocess studies that apply the measures to single therapy cases. These papers, contained in the sections, *Applications I: Process and Outcome* and *Applications II: Micro-Process*, include a review of research carried out on the referential process in Italy over the last decade (Mariani et al.); new results concerning the comparative effects of different forms of psychotherapy (Hong & Watson); a study of therapist interventions within the context of the Arousal function (Tocatly); an investigation of the psychological processes involved in disorders such as anorexia (Mariani et al.); the treatment of body dysmorphic disorder (Christian et al.); a multi-layered analysis of micro-process (Jaffe); and the comparative effects of in-person and remote treatment in psychoanalysis, including the first application of the new TDAAP (Peterson).

As we worked with the participating authors to bring their papers to print, this issue’s memorial character took on a more definite shape. As its subtitle implies, it is a tribute to the legacy of Bernard Maskit. But it is also a testament to the impressive scope of the research on the referential process and associated concepts that has been carried out over the last decade, not only in the United States and Canada, but also at three different research centers in Italy—at Bergamo, Rome, and Padua.

In marking the extent of the work that has been done, the papers gathered here also outline what was always important to Bernard Maskit—the work *yet to be done*. The development of the TDAAP was one of the last projects he was engaged in, and is described in some detail in his presentation. We expect to move forward with applications of this, and related programs, in our ongoing work, and expect that the introduction of time and associated nonsymbolic features of speech will reveal a more complex and multi-layered picture of the referential process as it plays out both within, and between individuals.

We hope that the papers in this issue not only demonstrate the breadth and scope of this multi-disciplinary international research program—combining theory, empirical measurement, and clinical observation—but inspire new work that will carry it even further.

## Data Availability

Data sharing is not applicable to this article as no datasets were generated or analyzed during the current study.

## Footnotes

1 We note that in this approach, Freud was following the same principle that was assumed by the developmental psychologists, Piaget (1950) and Bruner (1966); who identified several modes of thought but argued that mental development involved replacement of early or primitive modes with more advanced forms as the individual matures. Vygotsky (1986) viewed the functions of thought and speech as developing separately early in life and later joining, ‘whereupon thought becomes verbal and speech rational’, but he specifically excluded emotions from this intersection. From his perspective, expression of emotion began in primitive form and remained so throughout life.

2 When John Gedo, in his very prescient paper, referred to referential activity, our project was just beginning, and the broader scope of the referential process, with its several functions and measures, had not yet been developed. The paper of Charles Jaffe in this issue links the basic approach of Gedo and his colleagues to our current research measures.

3 To emphasize the contrast of this formulation with the classical view, we can examine its application to the interpretation of dreams. The classical view has been questioned by Erickson (1954) as applied to the ‘Irma’ dream. The application of the theory of the referential process to dreams, including Erickson’s formulation, has been discussed in detail by Bucci, 1997.

4 The term *tokens* refers to the number of individual words, counting multiplicity; the term *types* refers to the number of distinct individual words, not counting multiplicity.
